# Modulation of the colon cancer cell phenotype by pro-inflammatory macrophages: A preclinical model of surgery-associated inflammation and tumor recurrence

**DOI:** 10.1371/journal.pone.0192958

**Published:** 2018-02-20

**Authors:** María Marcuello, Xavier Mayol, Eloísa Felipe-Fumero, Jaume Costa, Laia López-Hierro, Silvia Salvans, Sandra Alonso, Marta Pascual, Luís Grande, Miguel Pera

**Affiliations:** 1 Colorectal Cancer Research Group, Cancer Research Program, IMIM (Hospital del Mar Medical Research Institute), Carrer Dr. Aiguader, Barcelona, Spain; 2 Section of Colon and Rectal Surgery, Department of Surgery, Hospital del Mar, Barcelona, Spain; National Cancer Center, JAPAN

## Abstract

Peritoneal infection after colorectal cancer surgery is associated with a higher rate of tumor relapse. We have recently proposed that soluble inflammatory factors released in response to a postoperative infection enhance tumor progression features in residual tumor cells. In an effort to set up models to study the mechanisms of residual tumor cell activation during surgery-associated inflammation, we have analyzed the phenotypic response of colon cancer cell lines to the paracrine effects of THP-1 and U937 differentiated human macrophages, which release an inflammatory medium characteristic of an innate immune response. The exposure of the colon cancer cell lines HT-29 and SW620 to conditioned media isolated from differentiated THP-1 and U937 macrophages induced a mesenchymal-like phenotypic shift, involving the activation of in vitro invasiveness. The inflammatory media activated the β-catenin/TCF4 transcriptional pathway and induced the expression of several mesenchymal (e.g., FN1 and VIM) and TCF4 target genes (e.g., MMP7, PTGS2, MET, and CCD1). Similarly, differential expression of some transcription factors involved in epithelial-to-mesenchymal transitions (i.e. ZEB1, SNAI1, and SNAI2) was variably observed in the colon cancer cell lines when exposed to the inflammatory media. THP-1 and U937 macrophages, which displayed characteristics of M1 differentiation, overexpressed some cytokines previously shown to be induced in colorectal cancer patients with increased rates of tumor recurrence associated with postoperative peritoneal infections, thus suggesting their pro-tumoral character. Therefore, the environment created by inflammatory M1 macrophages enhances features of epithelial-to-mesenchymal transition, and may be useful as a model to characterize pro-inflammatory cytokines as putative biomarkers of tumor recurrence risk.

## Introduction

Surgery is at present the only treatment with curative intent for patients with colorectal cancer (CRC) [1]. Still, operated CRC recurs in up to 40% of patients despite complete resection of the tumor. Among several prognostic factors, tumor stage and postoperative complications have the most negative impact on the oncological outcome [2–5]. In particular, anastomotic leakage after CRC surgery occurs at a frequency between 3 to over 15%, depending mostly on the tumor location [6]. Several studies have shown that the anastomotic leakage and subsequent intra-abdominal infections are associated with higher rates of tumor recurrence and cancer-specific mortality [7–17]. Indeed, the severity of the postoperative infection has also been correlated with the increased risk of recurrence [17, 18].

The association between postoperative systemic inflammation and tumor recurrence suggests that soluble factors released by the inflammatory response might stimulate residual cancer cells present in the surgical field, venous blood, and occult micrometastases. We have previously demonstrated an increased expression of circulating pro-inflammatory and pro-angiogenic factors in response to infection [14, 19, 20]. Our hypothesis was that these soluble molecules might facilitate the survival and growth of residual tumor cells in their path to recurrence. Direct evidence supporting this notion came from a murine model of colon cancer, where we demonstrated that the occurrence of a postoperative infection enhanced neovascularization of recurrent tumors [19]. More recently, by using CRC cell line-based in vitro assays, we were able to detect pro-invasion activities differentially present in the serum and peritoneal liquid samples from CRC patients with a postoperative course complicated with an intra-abdominal infection [13]. The presence of such soluble activities also suggested that the acquisition of tumor progression features by residual cancer cells is part of the mechanism of enhanced tumor recurrence in patients with postoperative complications.

The components of the acute inflammatory response that favor tumor recurrence remain elusive. Ascertaining whether particular soluble factors, or combinations of them, are responsible for the increased recurrence rate after surgery may lead to useful prognostic biomarkers. In this respect, we recently performed global gene expression analysis in circulating leukocytes from CRC patients undergoing surgery complicated with anastomotic leak and intra-abdominal infection [21]. The results revealed that several secreted cytokines, as part of the infection-induced inflammatory response, also had an involvement in cancer progression promoting processes such as invasiveness, angiogenesis, resistance to apoptosis, and immunoevasion. The validation of such molecules as serum markers to predict the risk of tumor recurrence should be of great help in the follow up after CRC surgery.

In vitro functional assays may help to validate biomarker candidates and, importantly, may improve our understanding of the functional significance of predictive biomarkers. Assays may be designed to characterize tumor subgroups specifically sensitive to the effects of certain cytokines. In this regard, we were able to set up cell-based in vitro functional assays using serum and peritoneal liquid samples from operated CRC patients [13, 20]. These experiments revealed that the fluid samples from patients undergoing postoperative infections contained biological activities that enhanced in vitro angiogenesis as well as the invasiveness of colon cancer cell lines. Amid a pro-angiogenic environment, the acquisition of an invasive phenotype by residual cancer cells might be a determinant start in their progression to recurrent tumors. Therefore, cell-based in vitro functional assays should expedite the identification of tumor features associated with the response to pro-tumoral inflammatory biomarkers.

In this report, we describe an in vitro model to study the cancer cell response to soluble cytokines mimicking the influence of an inflammatory response to surgery on residual tumor cells. We have used two human monocytic leukemia cell lines, THP-1 and U937, which can be differentiated to macrophages that produce a set of cytokines characteristic of an innate immune response [22, 23]. Conditioned media taken from these macrophages may be reminiscent of the environment present in patients who present an intra-abdominal infection after surgery. This model may allow to better delineate the identification of pro-inflammatory biomarkers predictive of tumor recurrence.

## Materials and methods

### Cell culture

The human monocytic leukemia THP-1 [24] and U937 [25] cell lines were obtained from the ECACC repository (catalogue numbers 88081201 and 85011440, respectively), and the human colon cancer cell lines HT-29 [26] and SW620 [27] were obtained from the ATCC repository (catalogue numbers HTB-38 and CCL-227, respectively). All the cell lines were preserved by the Cell Culture Maintenance Unit of the IMIM (Hospital del Mar Medical Research Institute) Core Facilities. Cells were grown under standard cell culture practices in complete Dulbecco's modified Eagle medium supplemented with 10% foetal bovine serum (DMEM+FBS), at 37°C, in a 5% CO_2_ atmosphere. Adherent cells were passaged twice weekly with trypsin using a seeding density of 2x10^4^ cells/cm^2^. THP-1 and U937 cells were grown in suspension and passaged twice weekly by dilution using a seeding density of 3x10^5^ cells/ml for THP-1 and 1,2x10^6^ cells/ml for U937. Macrophage differentiation was induced according to Satsu *et al*. [22]. Briefly, cells were seeded at the latter density using 3 ml/cm^2^ DMEM+FBS containing 200 nM phorbol-12-myristate-13-acetate (PMA), and left incubating for 4 days. Afterwards, culture flasks were washed with DMEM+FBS twice, and incubated 24 hours with fresh DMEM-FBS to produce the inflammatory conditioned medium (MACRO-CM). As controls, THP-1 cells were incubated in parallel in the absence of PMA to produce non-inflammatory conditioned medium (MONO-CM), and DMEM+FBS was incubated without cells to produce control media (CON). In some experiments, medroxyprogesterone (MPA) was used to induce a M1-to-M2 phenotypic switch on differentiated macrophages [28]. After PMA differentiation, cells were further treated with 0.1 μM MPA (MPA) or left untreated (MPA-CON) for 24 hours. After incubation, cells were lysed for RNA analysis.

### Cell proliferation assays

To measure the kinetics of cell proliferation, HT-29 and SW620 cells were seeded in 24-well plates at 2x10^4^ cells/cm^2^. The day after seeding, the culture medium was replaced by the conditioned media to initiate a continuous course of treatment. At the indicated periods of time, cell suspensions from duplicate wells were obtained by trypsin digestion and cells counted under the microscope by trypan blue exclusion.

### Cell clonogenicity assays

The clonogenic capacity of cells after treatment with conditioned media was analyzed as a measure of cell viability. After treatment for 48 hours, cell suspensions were obtained by trypsin digestion, and the totality of cells present in the plate was counted under the microscope using a Neubauer chamber. Cells were then seeded on 57 cm^2^ plates at a clonogenic density (10 cells/cm^2^) and allowed to grow in standard DMEM+FBS medium. After 10 days, plates were stained with coomassie blue and blue colonies counted under the naked eye.

### Wound healing assays

To measure the migratory capacity of cells, HT-29 cells were seeded at 2x10^4^ cells/cm^2^ on petri dishes and allowed to grow until homogenous cell confluence was reached. Then, a rounded plastic tip was used to scrap several lanes on the cell monolayer to draft a grid devoid of cells. Plates were washed twice with standard DMEM+FBS medium and finally incubated with conditioned media or control media for 24 and 48 hours. Several crossings of the scrapped grid were marked at the beginning of each treatment condition, so that sequential images of each crossing were taken during the course of the treatment, and the healing distance was calculated on them.

### Migration and invasion assays using transwells

Transwell assays were carried out as described previously [13]. Briefly, cells were incubated without FBS overnight, disaggregated with Accutase (Life Technologies, UK) and seeded on 96-transwell permeable supports with 8μm pore size (Corning Life Sciences, USA). In invasion assays, transwells were covered with 30 μl of a Basement Membrane Extract solution poured the day before seeding (Trevigen, USA). Conditioned media were split in 96-well receiver plates and the transwell supports placed on these plates. Migration and invasion were allowed to proceed in the cell culture incubator for 24 hours. The relative number of cells migrating or invading through the transwells were estimated by using the hexosaminidase cell counting assay [29].

### Luciferase reporter transcriptional assays

Transfections were performed using Lipofectamine LTX with Plus Reagent (Life Technologies, UK). Subconfluent cell cultures were incubated with the mix of DNA and transfection reagents for 4 hours, the media changed to conditioned media as indicated, and luciferase activty analyzed after 24 hours of additional culture using the Dual Luciferase Reporter Assay System (Promega, USA). Luciferase reporter plasmids were pTOPGLOW and pFOPGLOW that contain the luciferase reporter gene under the control of TCF4-responsive, DNA-binding elements as wild type and mutated versions, respectively [30]. The normalizer plasmid was pRL-SV40 that contains the renilla luciferase cDNA under the control of a constitutive viral gene promoter (Promega, USA).

### RNA isolation and Real Time PCR

Total RNA was isolated using the GenElute Mammalian Total RNA Kit (Sigma, USA) following the manufacturer instructions, including further DNAse I digestion with the DNA-free Kit (Ambion, USA) to remove traces of genomic DNA. cDNA was obtained using the TaqMan Reverse Transcription Reagents kit (Aplied Biosystems, USA). Relative quantification of cDNA was performed by real time polymerase chain reaction (RT-PCR) using the Power SYBR Green PCR Master Mix (Applied Biosystems, USA). Primer sequences are shown in Table 1. RT-PCR results were normalized with the 18S rRNA as a housekeeping gene and expressed as relative mRNA quantification by using the 2-ΔΔCT method [31].

**Table 1:** Primer sequences used in RT- PCR assays.

Gene	Forward primer (5’ to 3’)	Reverse primer (5’ to 3’)
18S	CGGCGGCTTTGGTGACTCT	ATGGTAGGCACGGCGACTA
IL6	TGAAAGCAGCAAAGAGGCAC	CAGGAACTGGATCAGGACTTTTG
IL1B	GGGACAGGATATGGAGCAACA	CATCTTTCAACACGCAGGACA
CXCL10	GCCATTCTGATTTGCTGCCTTA	GATTCAGACATCTCTTCTCACCCTTC
CD163	GAAGAAGCCAAAATTACCTGCTCA	AGAGAGAAGTCCGAATCACAGATG
IL10	CGAGATGCCTTCAGCAGAGTG	CGCCTTGATGTCTGGGTCTTG
FN1	ACCAATGCCAGGATTCAGAGA	TGATAAATACTTCGACAGGACCACT
VIM	CCAGGCAAAGCAGGAGTC	GTTCAACGGCAAAGTTCTCTTC
ITGB6	ACCAGAAGAAATTGCCAACCCT	GAGTCATTCCGCCAGCCA
S100A4	GGCAAAGAGGGTGACAAGTTC	GATGCAGGACAGGAAGACAC
ZEB1	GACATCACATAAATCAGGAAGAGATCA	TCGCCCATTCACAGGTATCA
SNAI1	CCAATCGGAAGCCTAACTACAG	ACAGAGTCCCAGATGAGCAT
SNAI2	TCAAGGACACATTAGAACTCACAC	ACAGCAGCCAGATTCCTCA
TWIST	CAGCTATGTGGCTCACGAG	GACTGTCCATTTTCTCCTTCTCTG
MMP7	CATGAGTGAGCTACAGTGGGAAC	GCATCTCCTTGAGTTTGGCTTC
PTGS2	TATACTAGAGCCCTTCCTCCTGTG	TGTCTAGCCAGAGTTTCACCGTA
MET	TGTCTAGCCAGAGTTTCACCGTA	CAATCACTTCTGGAGACACTGGAT
CCD1	CGGAGGAGAACAAACAGATCATC	GCGGTAGTAGGACAGGAAGT
FGFBP2	CCGAGGGTGACAGGTGAAAGA	CGTTGGATTGAAAGCGGCATA
MMP9	CAACTGTCCCTGCCCGAGA	GCAAAGGCGTCGTCAATCAC
IL1R2	TGCTGGAGGTGAAAGTCTGG	TGAAGGGTGAAGGCAGAAACTC
PLSCR1	TGGAATTTTGAGAGAGGCATTTACA	TCCTGATTTTTGTTCCTGGCTG
TRPS1	ATTGCCTGACCACAAAGACCT	GCCTGAAGTGCCTCTGGGTTA
ORM1	TTCAGGAGATCCAAGCAACCTTC	CAGTTCTTCTCATCGTTCACGTCA
HP	CTTCCAGAGGCAAGACCAACCA	TGCGATATCCGTGACATCATTG
ADAM9	CAACAGACCTCACATCTTTCTTCTTATGA	GATGAATTATGAACTCCCTCCACATAG
PDGFC	GGCGGAATCCAACCTGAGTAGTA	ATGAGGAAACCTTGGGCTGTGA

### Statistical analysis

Quantitative data was analyzed by means of the Wilcoxon sign-rank tests where 4 or more independent experiments were performed. Statistical analysis was performed using STATA version 15 (STATA Corp., Texas, USA).

## Results

**Fig 1:**
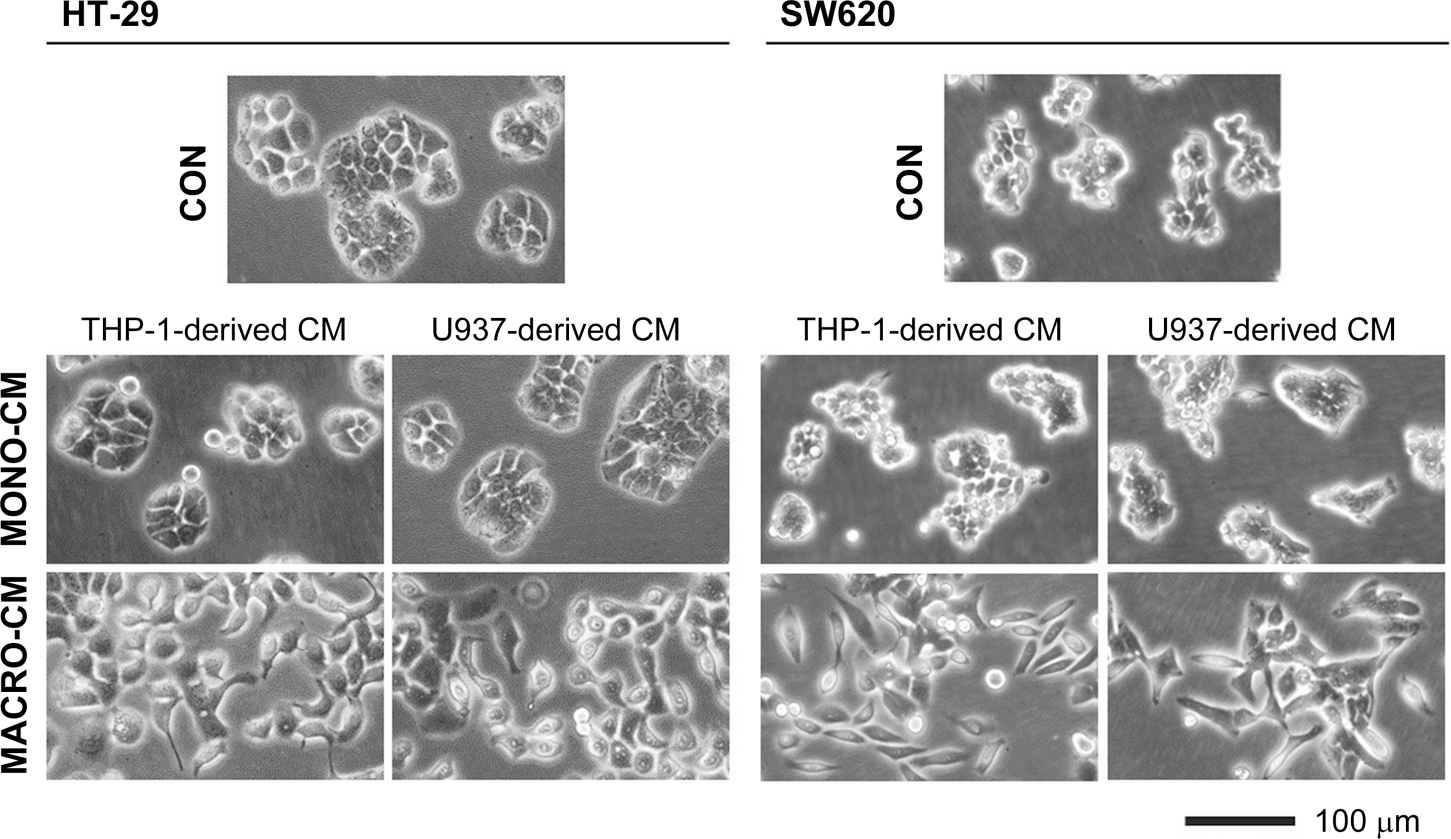
Loss of epithelial morphology. The inflammatory medium conditioned by macrophages induced a morphological shift in the epithelial colon cancer cell lines HT-29 and SW620 that was reminiscent of an epithelial-to-mesenchymal transition. Phase contrast microscopy images were obtained after treatment of colon cancer cell cultures for 2 days with the indicated conditioned media. *CON*, standard medium; *MONO-CM*, conditioned medium from non-activated monocytes; *MACRO-CM*, conditioned medium from differentiated macrophages.

**Fig 2:**
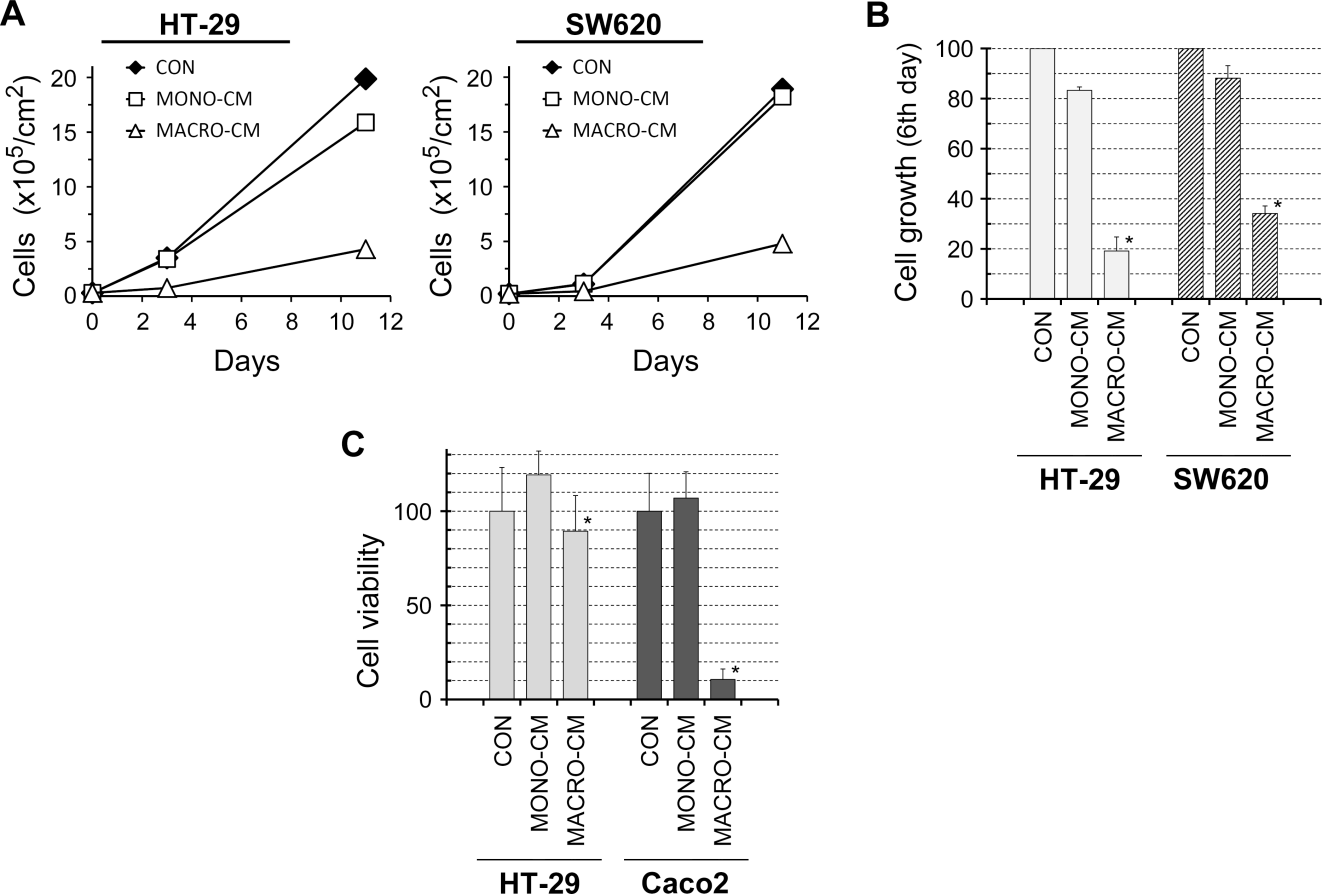
Cell line-dependent cell proliferation and toxicity. The inflammatory medium conditioned by macrophages provoked a slowdown of cell proliferation in the HT-29 and SW620 cell lines, whereas it induced cell death in Caco2 cells. ***A***, cell proliferation assays on HT-29 and SW620 during continuous treatment with the indicated conditioned media. Cell cultures were seeded at 3x10^4^/cm^2^ and 2x10^4^/cm^2^, respectively, and viable cells were calculated by trypan blue exclussion during the course of continuous treatment. The graph shows a representative experiment made in duplicates. ***B***, cell countings were performed on the sixth day of the experiment shown in *A* and are expressed as the mean ± SD from 4 independent experiments (*, p = 0.068 with respect to *CON* or *MONO-CM* according to the Wilcoxon sign-rank test). ***C***, cell viability was analyzed by clonogenicity assays as described in Materials and Methods, revealing that HT-29 cells preserved almost full viability after treatment with MACRO-CM for 48 hours while Caco2 cells underwent massive cell death. Results are shown as the percentage of visible colonies respect to 100% viability in cultures that had been treated with standard medium (*CON*). Percentage values are the mean ± SD from 6 independent experiments (*, p<0.050 with respect to *CON* or *MONO-CM* according to the Wilcoxon sign-rank test). *CON*, standard medium; *MONO-CM*, conditioned medium from non-activated monocytes; *MACRO-CM*, conditioned medium from differentiated macrophages.

We searched for macrophagic cell lines with features characteristic of an innate immune response to set up in vitro models that reproduce the mechanisms of tumor progression driven by inflammatory processes–e.g. peritoneal infections after colorectal cancer surgery. The human leukemia cell lines THP-1 and U937 had been previously used to produce activated macrophages that secreted a M1-type cytokine pattern [22,23]. We thus used these cell lines to investigate whether macrophage conditioned media exerted any effects on the tumor progression features of colon cancer cell lines. Treatment of the colon cancer cell lines HT-29 and SW620 with conditioned media from PMA-differentiated THP-1 or U937 macrophages (MACRO-CM) led to a dramatic change in the cell phenotype compared to treatment with conditioned media from non-differentiated monocytic cells (MONO-CM) or control media (CON). Both colon cancer cell lines lost their epithelial appearance in the presence of MACRO-CM to acquire a more flatten, less adhesive morphology that led to cell scattering (Fig 1). This phenotypic shift was accompanied by a slowdown of cell proliferation during the continuous treatment with MACRO-CM (Fig 2A and 2B). The lower rate of cell proliferation was not due to cell death because cell viability was fully preserved in clonogenic assays (Fig 2C). In contrast to HT-29 and SW620, another colon cancer cell line, Caco2, underwent significant toxicity and cell death just after 48 hours of treatment (Fig 2C), as previously reported [22].

**Fig 3:**
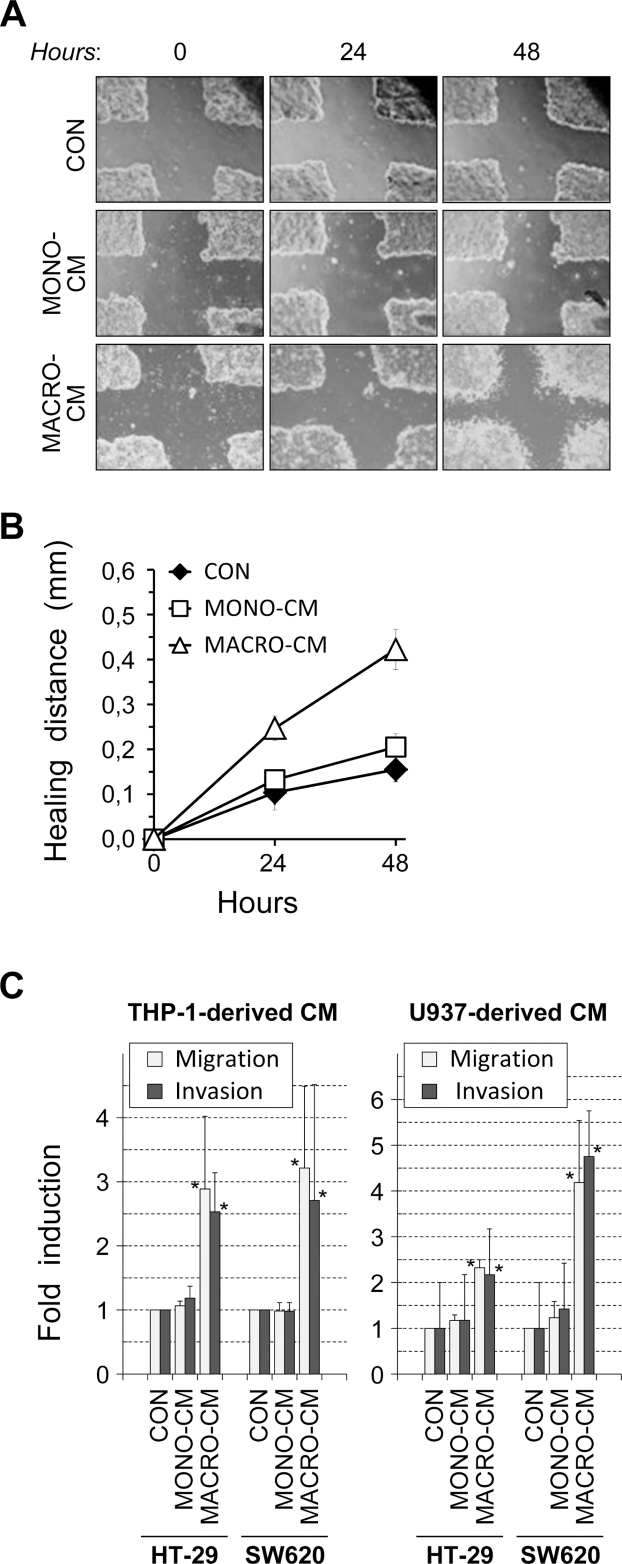
In vitro cell migration and invasion. The inflammatory medium conditioned by macrophages induced in vitro migration and invasion activities in colon cancer cells. ***A***, wound healing assays using the HT-29 cell line. Confluent monolayers of HT-29 cell cultures were scrapped with a rubber tip (*0 hours*) and thereafter treated with the indicated conditioned media for 48 hours. Cell scattering and increased healing migration were apparent after 48 hours of treatment with MACRO-CM. ***B***, images of crossed wounds were taken after 24 and 48 hours of continuous treatment in the experiment shown in ***A***, and the closing distances near the cross corners were measured. The graph shows the mean (mm) ± error of two independent experiments, each one integrating an average of the healing distances of several measures in each cell monolayer. ***C***, in vitro migration and invasion assays were performed using HT-29 or SW620 cells cultured on transwells for 24 hours in the presence of the indicated conditioned media. Results are the mean ± SD of cells moving through the transwell relative to the basal migration or invasion using standard medium as a control (*CON*) from at least 6 independent experiments in each culture condition. (*, p<0.03 with respect to *CON* or *MONO-CM* according to the Wilcoxon sign-rank test). *CON*, standard medium; *MONO-CM*, conditioned medium from non-activated monocytes; *MACRO-CM*, conditioned medium from differentiated macrophages.

The morphological shift observed in HT-29 and SW620 cells was reminiscent of an epithelial-to-mesenchymal transition (EMT), a phenomenon that involves the acquisition of migratory and invasive properties by cancer cells of epithelial origin. To test this possibility, we first performed in vitro wound-healing assays on confluent cultures of HT-29 cells. The capacity of HT-29 cells to repair cell monolayer wounds was greatly enhanced by treatment with THP-1-derived MACRO-CM in comparison to MONO-CM or control medium (Fig 3A and 3B). Moreover, we performed in vitro migration and invasion assays using transwells. The results again showed that both migration and invasion activities were significantly induced in the presence of MACRO-CM obtained from the two macrophage cell lines THP-1 or U937 (Fig 3C).

**Fig 4:**
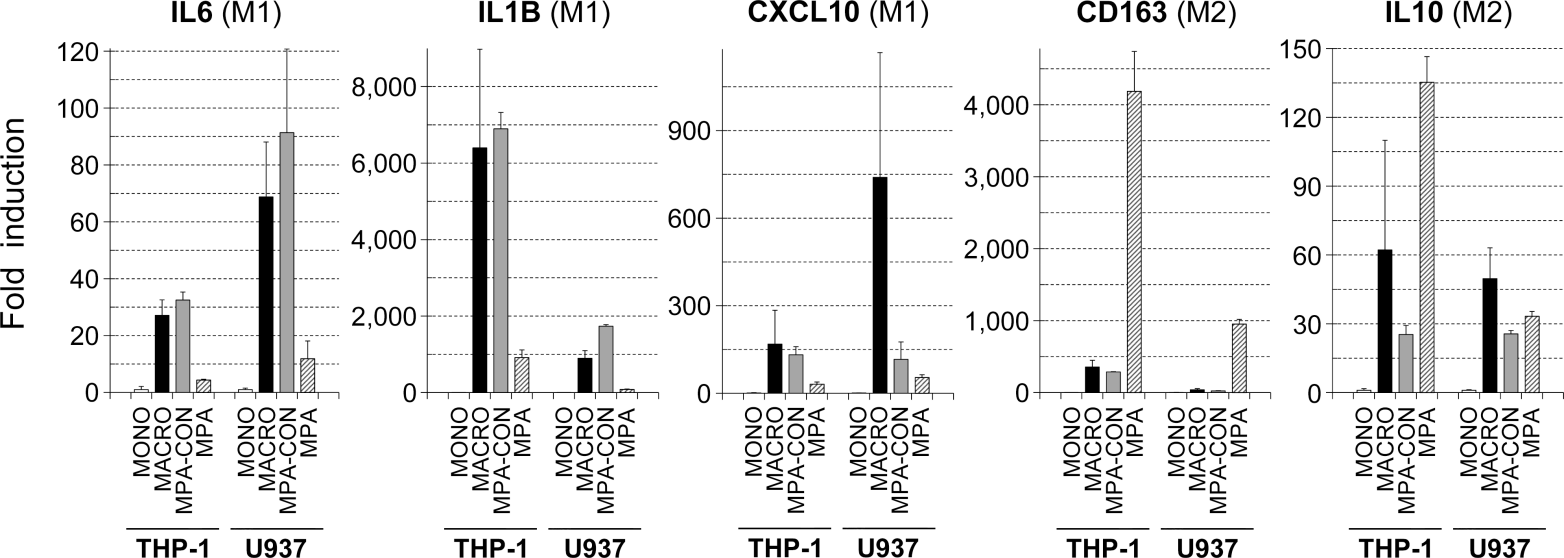
PMA-differentiated THP-1 and U937 macrophages displayed features of a M1 type phenotype. PMA-differentiated THP-1 and U937 macrophages (*MACRO*) were harvested in parallel with non-treated cultures (*MONO*), and total RNA isolated. Some cultures were further incubated in the presence of medroxyprogesterone (*MPA*) for 24 hours or left untreated (*MPA-CON*), and similarly harvested and total RNA isolated. RNA samples were reverse transcribed to cDNA, and Real Time PCR analysis was performed using oligonucleotide primers specific for the indicated genes (see Material and Methods). Markers of the M1 phenotype (*IL6*, *IL1B*, *CXCL10*) displayed strong induction during PMA-differentiation but were abruptly suppressed with further incubation with the M2 inducer MPA. In contrast, induction of the M2 markers CD163 and IL10 was modest, and in the case of CD163 potently enhanced by the M2 inducer MPA. Results are shown as the relative levels of each mRNA in the differentiated cell lines (*MACRO*, *MPA-CON*, *MPA*) respect to the non-treated monocytic cell lines (*MONO*) and expressed as the mean ± error of at least 2 independent experiments.

The results above indicated that the inflammatory media produced by THP-1 or U937 macrophages enhanced the invasiveness of colon cancer cells by inducing an EMT-like phenotype. Similar phenomena is known to occur during the course of in vivo tumor progression when tumor cells recruit infiltrating macrophages, the so called tumor-associated macrophages (TAMs). TAMs are initially induced as part of the innate immune response to cancer cells but display plasticity in their differentiation capabilities: by responding to tumor cell-derived signals TAMs can polarize from a pro-inflammatory M1 type into a pro-tumoral M2 type of differentiation that contributes to tumor progression [32]. Since THP-1 and U937 cell cultures differentiated by PMA display a pattern of cytokines consistent with pro-inflammatory macrophages or M1-type [22, 23], our results suggest that the protumoral macrophagic activity is not an exclusive feature of TAMs, but that M1 macrophages can also exert protumoral activities. We thus analyzed the expression of M1 and M2 markers in our differentiated THP-1 and U937 macrophages to confirm whether they displayed a M1 phenotype. We found that the expression of cytokine genes associated with the M1 phenotype (IL6, IL1B, CXCL10) were strongly induced in PMA-differentiated macrophages from the two macrophagic cell lines, while markers of the M2 phenotype (CD163, IL10) were induced at limited levels (Fig 4). Moreover, we used medroxyprogesterone (MPA) to further confirm the M1 character of the macrophagic phenotype obtained in our experiments. Recently, Tsai et al. [28] showed that MPA induced a M1-to-M2 differentiation shift in THP-1 macrophages. We also used a similar approach to show that the induction of M1-associated genes induced in PMA-activated THP-1 and U937 macrophages (IL6, IL1B, CXCL10) was abolished by MPA, while the expression of the M2-associated cytokine CD163 was potently induced by MPA (Fig 4). Together, these results supported the occurrence of the M1 differentiation phenotype in our PMA-activated THP-1 and U937 macrophages and implicate this M1 phenotype in the protumoral activity observed on the colon cancer cell lines.

**Fig 5:**
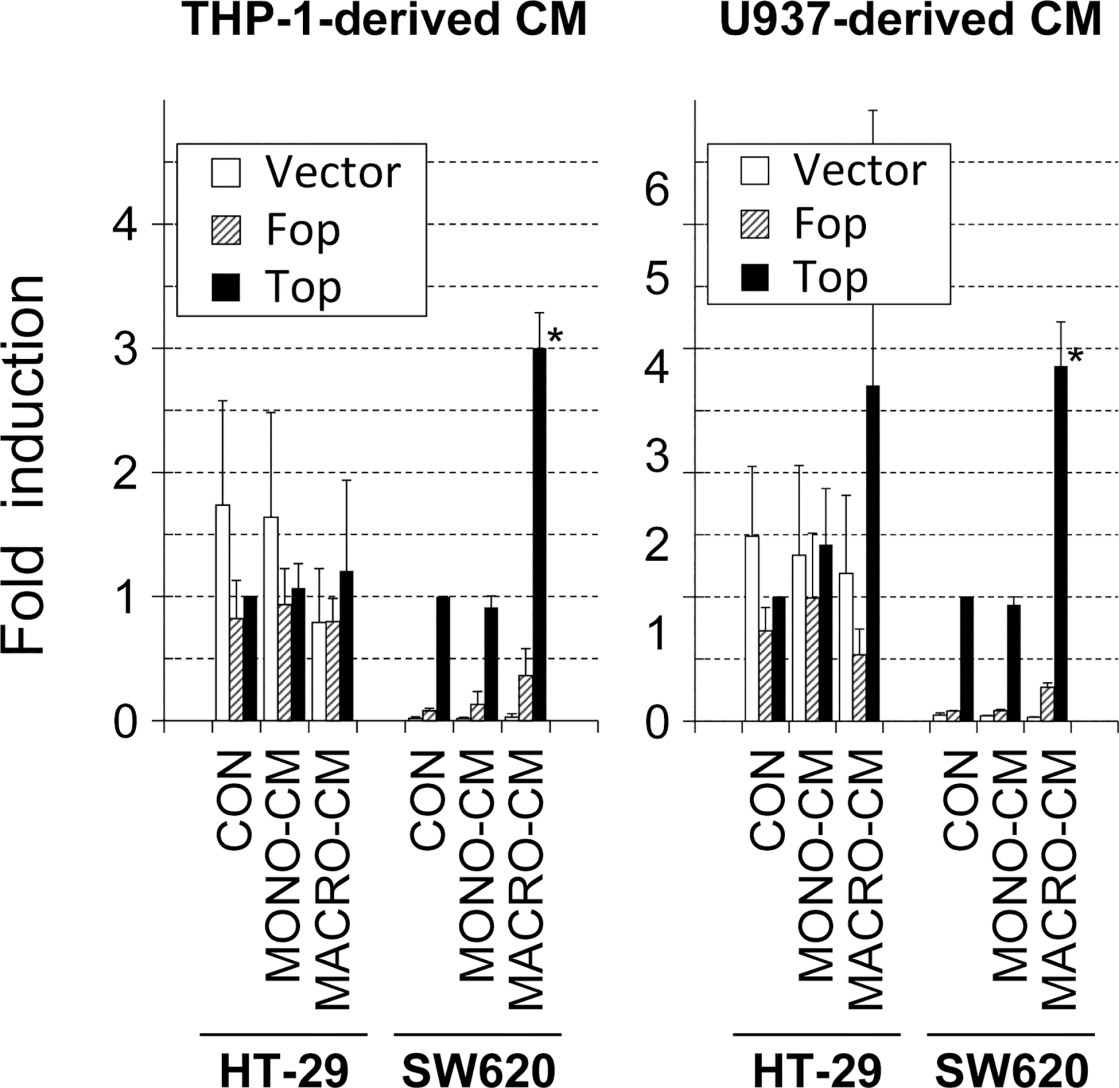
β-catenin/TCF4 transcriptional activity. The inflammatory media conditioned by THP-1 or U937 macrophages induced the activity of the transcription factor β-catenin/TCF4 in SW620 colon cancer cells. Cultures of HT-29 or SW620 cells were transfected with the Firefly luciferase reporter plasmid *Top*, which contains an artificial gene promoter with TCF4-responsive DNA binding sites. As controls, the vector containing the TCF4 DNA binding sites mutated (*Fop*) and the empty vector (*vector*) were transfected in parallel. After 4 hours of transfection, cell cultures were treated with the indicated conditioned media for 24 hours and finally harvested. Results are shown relative to the TOP activity of cells treated with standard medium (*CON*) and expressed as the mean ± SD of at least 3 independent experiments (*, p = 0.068 with respect to *CON* or *MONO-CM* according to the Wilcoxon sign-rank test). *CON*, standard medium; *MONO-CM*, conditioned medium from non-activated monocytes; *MACRO-CM*, conditioned medium from differentiated macrophages.

To get insight into the mechanisms underlying the invasive phenotype shift, we next performed experiments to analyze some known mediators of cancer cell invasiveness. During tumor progression, and especially during EMT, the protein β-catenin is displaced from cell-to-cell adhesion complexes and translocates to the nucleus. In the nucleus, β-catenin dimerizes with TCF4 to form a transcription factor that may activate a mesenchymal-like gene expression program. We thus tested whether the transcriptional activity of β-catenin/TCF4 was induced by treating the colon cancer cell lines with MACRO-CM. In the case of SW620 cells, the transcriptional activity of a transiently transfected β-catenin/TCF4 luciferase reporter was induced 3-fold, approximately, by treatment with MACRO-CM from both THP-1 and U937 compared to treatment with MONO-CM or control media (Fig 5). In the case of HT-29 cells, the induction of the β-catenin/TCF4 luciferase reporter by MACRO-CM from THP-1 macrophages was not observed, and a modest increase in this activity using MACRO-CM from U937 macrophages was not statistically significant (Fig 5). Therefore, the invasive phenotype induced by inflammatory media from macrophages implicated the activation of the β-catenin/TCF4 transcriptional pathway in at least some cancer cells.

**Fig 6:**
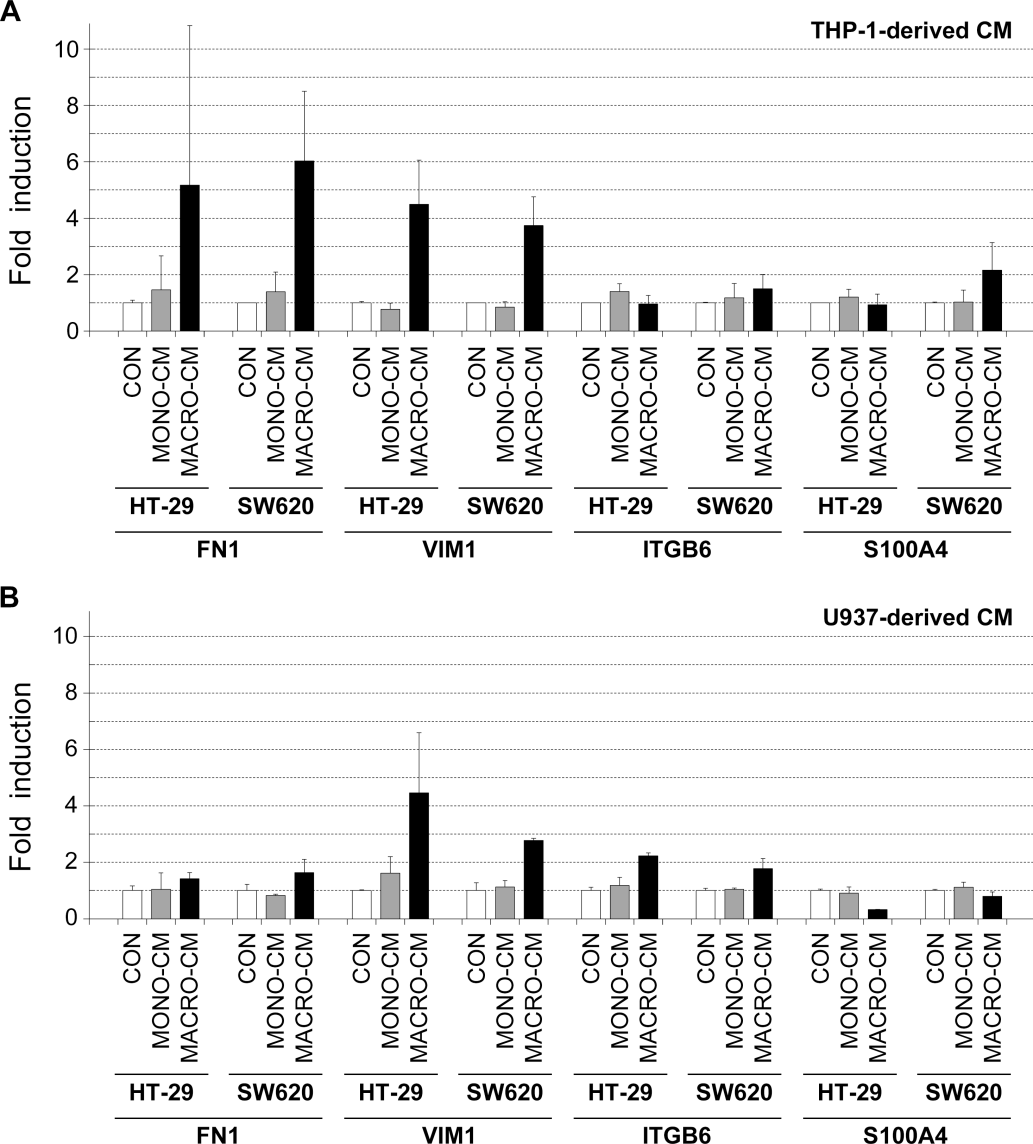
Gene expression analysis of EMT-related markers. The inflammatory medium conditioned by THP-1 or U937 macrophages induced the expression of several genes related to the EMT process. Total RNA was isolated from HT-29 or SW620 cell cultures that had been treated with the indicated conditioned media for 48 hours. RNA samples were reverse transcribed to cDNA, and Real Time PCR analysis was performed using oligonucleotide primers specific for the indicated genes (see Material and Methods). Genes analyzed were: *FN1* (fibronectin 1), *VIM* (vimentin), *ITGB6* (integrin subunit beta 6), and *S100A4* (S100 calcium binding protein A4). Results are shown as the relative levels of each mRNA respect to the sample treated with standard medium (*CON*) and expressed as the mean ± SD of at least 3 independent experiments. *CON*, standard medium; *MONO-CM*, conditioned medium from non-activated monocytes; *MACRO-CM*, conditioned medium from differentiated macrophages.

We next used RT-PCR analysis to analyze the expression of several genes known to be regulated during EMT to further characterize the invasive phenotype induced by the inflammatory media. First, we analyzed some mesenchymal markers not expressed by epithelial cells but commonly induced during colon cancer EMT. We found that the mRNA levels of the extracellular matrix protein fibronectin (FN1) and of the mesenchymal intermediate filament vimentin (VIM) were potently induced in both HT-29 and SW620 cells in the presence of MACRO-CM from both THP-1 and U937 macrophages (Fig 6), except that only a modest increase in fibronectin mRNA was observed when using conditioned media from U937 macrophages. Moreover, other mesenchymal markers displayed variable expression patterns depending on the culture condition. The integrin subunit beta 6 protein mRNA (ITGB6) was induced in the two colon cancer cell lines by treatment with MACRO-CM from U937 macrophages, and the colon cancer metastasis-related protein S100A4 mRNA was induced in SW620 cells by treatment with THP-1 MACRO-CM (Fig 6). These results confirmed that the colon cancer cell lines acquired mesenchymal features when treated with inflammatory media produced by macrophages despite differences in the induction of particular mesenchymal markers among the diverse culture conditions.

**Fig 7:**
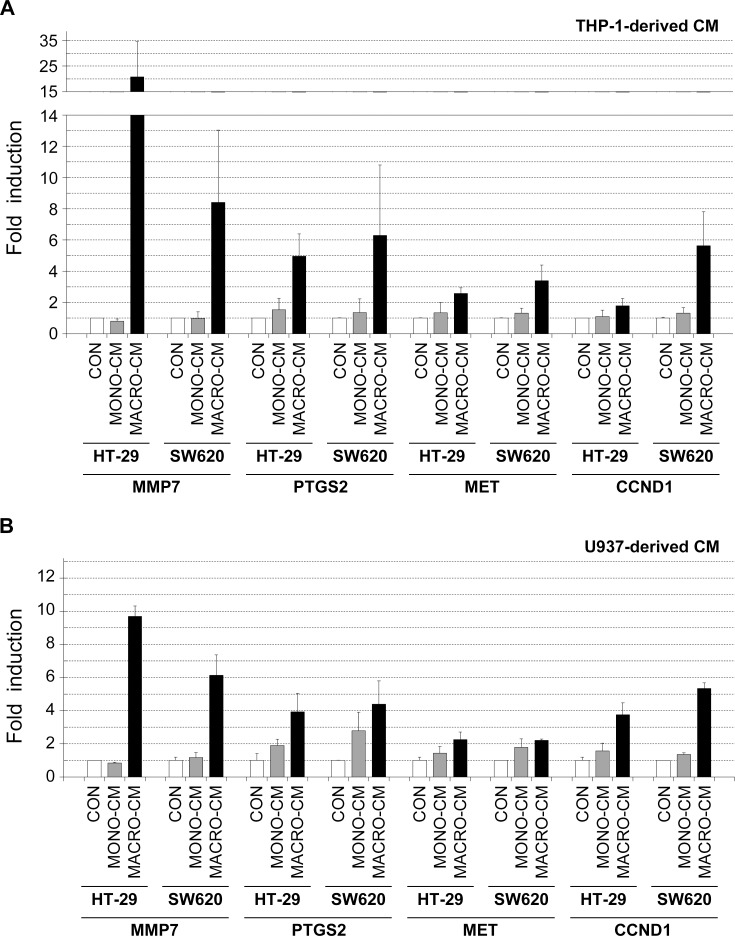
Gene expression analysis of β-catenin/TCF4 transcriptional target genes. The inflammatory medium conditioned by THP-1 or U937 macrophages induced the expression of some targets of the β-catenin/TCF4 transcriptional pathway. Total RNA was isolated from HT-29 or SW620 cell cultures that had been treated with the indicated conditioned media for 48 hours. RNA samples were reverse transcribed to cDNA, and Real Time PCR analysis was performed using oligonucleotide primers specific for the indicated genes (see Material and Methods). Genes analyzed were: *MMP7* (matrix metallopeptidase 7), *PTGS2* (prostaglandin-endoperoxide synthase 2, or COX-2), *MET* (MET proto-oncogene, receptor tyrosine kinase), and *CCND1* (cyclin D1). Results are shown as the relative levels of each mRNA respect to the sample treated with standard medium (*CON*) and expressed as the mean ± SD of at least 3 independent experiments. *CON*, standard medium; *MONO-CM*, conditioned medium from non-activated monocytes; *MACRO-CM*, conditioned medium from differentiated macrophages.

Second, we analyzed the expression of some β-catenin transcriptional target genes as the previous β-catenin/TCF4 transcriptional reporter assays suggested that this pathway was activated during the invasive phenotype shift induced by inflammatory media. The results showed that the mRNA levels of the matrix metallopeptidase 7 (MMP7), the prostaglandin-endoperoxide synthase 2, also known as COX-2 (PTGS2), the oncogenic growth factor receptor MET, and the cell cycle protein cyclin D1 (CCND1) were clearly induced in the two colon cancer cell lines by MACRO-CM from both THP-1 and U937 macrophages (Fig 7). We also analyzed the expression of other targets of the β-catenin/TCF4 pathway and found that they were induced by inflammatory media in a particular colon cancer cell line or not induced (see raw data in the supplementary file S1 Fig). These results, together with the transcriptional reporter assays shown in Fig 5, were thus consistent with the involvement of the β-catenin/TCF4 pathway in the enhancement of tumor progression features of colon cancer cell lines by inflammatory media from macrophages.

**Fig 8:**
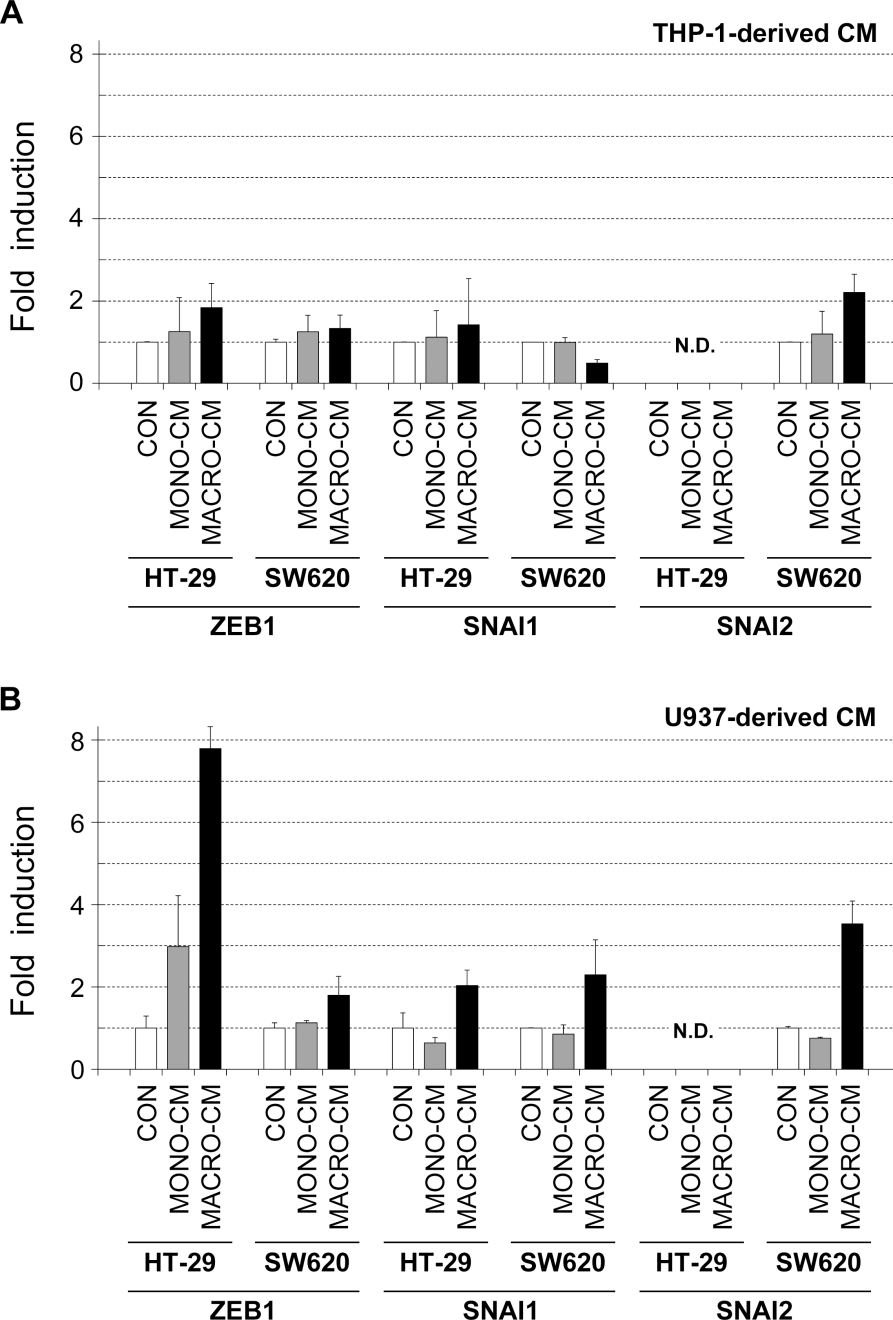
Gene expression analysis of EMT-related transcription factors. The inflammatory medium conditioned by THP-1 or U937 macrophages induced the expression of some transcription factor genes known as mediators of the EMT process. Total RNA was isolated from HT-29 or SW620 cell cultures that had been treated with the indicated conditioned media for 48 hours. RNA samples were reverse transcribed to cDNA, and Real Time PCR analysis was performed using oligonucleotide primers specific for the indicated genes (see Material and Methods). Genes analyzed were: *ZEB1* (zinc finger E-box binding homeobox 1), *SNAI1* (snail family transcriptional repressor 1, or Snail), and *SNAI2* (snail family transcriptional repressor 2, or Slug). Results are shown as the relative levels of each mRNA respect to the sample treated with standard medium (*CON*) and expressed as the mean ± SD of at least 3 independent experiments. *N*.*D*., not detectable; *CON*, standard medium; *MONO-CM*, conditioned medium from non-activated monocytes; *MACRO-CM*, conditioned medium from differentiated macrophages.

Third, we finally analyzed the expression of transcription factors known to be mediators of EMT in colon cancer cells, namely ZEB1, SNAI1 (also known as Snail), SNAI2 (also known as Slug), and TWIST. We found that the expression of ZEB1, SNAI1 and SNAI2 were clearly induced in the two colon cancer cell lines by MACRO-CM from U937 macrophages, with the exception of HT-29 cells that did not expressed detectable levels of SNAI2 mRNA under any culture condition (Fig 8). With respect to MACRO-CM obtained from THP-1 macrophages, it provoked modest induction of SNAI2 mRNA in SW620 cells, as well as ZEB1 and SNAI1 in HT-29 cells (Fig 8). The mRNA levels of the TWIST transcription factor could not be detected in the colon cancer cell lines in any treatment condition. These results implicated the induction of EMT trascription factors as part of the colon cancer cell phenotype shift that occurs in the presence of inflammatory media.

**Fig 9:**
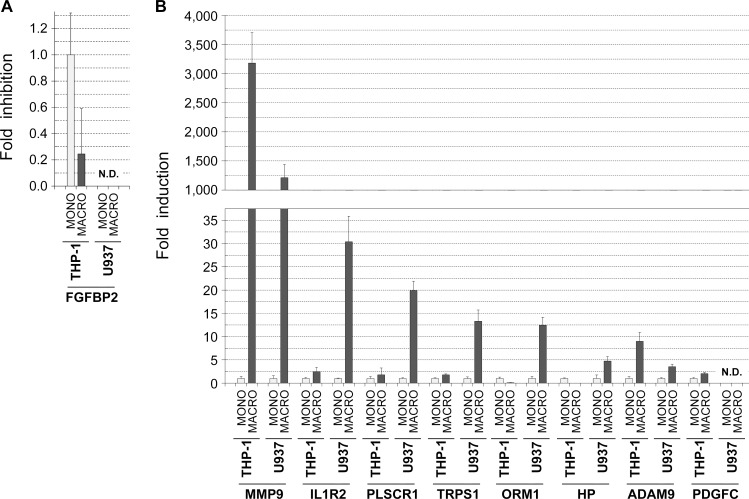
Cytokine genes induced by PMA-differentiated THP-1 and U937 macrophages suggested common mechanisms with the tumor recurrence in colorectal cancer patients undergoing postoperative peritoneal infections. PMA-differentiated THP-1 and U937 macrophages (MACRO) were harvested in parellel with non-treated cultures (MONO), and total RNA isolated. RNA samples were reverse transcribed to cDNA, and Real Time PCR analysis was performed using oligonucleotide primers specific for the indicated genes (see Material and Methods). Genes analyzed corresponded to a gene panel differentially expressed in peripheral blood leukocytes of colorectal cancer patients undergoing postoperative peritoneal infetions (see reference 14): *MMP9* (matrix metallopeptidase 9), *IL1R2* (interleukin 1 receptor type 2), *PLSCR1* (phospholipid scramblase 1), *TRPS1* (transcriptional repressor GATA binding 1), *ORM1* (orosomucoid 1), *HP* (haptoglobin), *ADAM9* (ADAM metallopeptidase domain 9), and *PDGFC* (platelet derived growth factor C). Results are shown as the relative levels of each mRNA in the PMA treated cell lines (*MACRO*) respect to the non-treated monocytic cell lines (*MONO*) and expressed as the mean ± SD of at least 3 independent experiments. *N*.*D*., not detectable.

Recently, we reported a gene expression analysis of cytokines produced by peripheral blood leukocytes in colon cancer patients that underwent postoperative peritoneal infections [14]. In that study, we proposed a panel of secreted proteins that would act as sistemic factors on cancer cells residual after surgery to promote tumor recurrence. Interestingly, we found that some of those proteins were also differentially expressed in the present in vitro model when the two macrophagic cell lines THP-1 and U937 were induced to differentiate with PMA. In particular, the mRNA levels of the matrix metallopeptidase 9 (MMP9) were strongly induced in both cel lines (Fig 9). Moreover, variable levels of induction were also observed when analyzing the mRNA of the genes IL1R2, PLSCR1, TRPS1, ORM1, HP, ADAM9, and PDGFC (Fig 9). Hence, these results suggested that the in vitro model described in the present study reproduces, at least in part, mechanisms of tumor progression similar to those occurring during the process of tumor recurrence in colorectal cancer patients undergoing postoperative peritoneal infections.

Summarizing, the inflammatory medium released by differentiated THP-1 or U937 macrophages led to a phenotypic shift in colon cancer cells with features of EMT, including increased cell invasiveness, activation of a gene expression program controlled by β-catenin/TCF4, and induction of mesenchymal markers and transcription factors genes related to the EMT process. Differentiated macrophages displayed a cytokine gene expression pattern indicative of a M1 phenotype indicating that protumoral macrophagic differentiation is not restricted to the tumor associated macrophages with a M2 phenotype. Of relevance, some of the cytokines produced overlap a gene expression panel previously found to be differentially expressed in peripheral blood leukocytes of colorectal cancer patients undergoing postoperative peritoneal infections.

## Discussion

Standardized preclinical models are important for uncovering the mechanisms of tumor progression and for the identification of cancer biomarkers. In this study, we have characterized the effects of THP-1 and U937 inflammatory macrophages on tumor progression features of colon cancer cells. We aimed at using this system to model some of the tumor recurrence mechanisms that take place in residual cancer cells after CRC surgery. Our results showed that some colon cancer cell lines with epithelial characteristics (i.e., HT-29 and SW620) underwent a phenotypic shift to a mesenchymal-like phenotype when treated with culture medium conditioned by inflammatory THP-1 and U937 macrophages. The acquired phenotype involved the enhancement of cell migration and invasion, reminiscent of EMT, together with the activation of the β-catenin/TCF4 transcriptional pathway and gene expression of some other EMT markers. These results hence reveal some tumor progression effects induced by soluble pro-inflammatory factors characteristic of the innate immune response, so that they might be putative drivers of tumor recurrence associated with postoperative infections.

The acquisition of invasive properties by exposure of colon cancer cell lines to the THP-1 and U937 macrophage conditioned medium was comparable to that observed previously by our group when studying the mechanisms of tumor recurrence after surgery with curative intent [13]. In those studies, samples from patients diagnosed with anastomotic leak and subsequent postoperative intra-abdominal infection were compared with samples from patients without postoperative complications in a series of in vitro assays. Among the results obtained, samples from infected patients yielded migration and invasion activities on cell lines significantly higher than samples from patients with uncomplicated postoperative courses.

In this context, the present in vitro model may be a suitable complement for the identification of pro-inflammatory markers predictive of tumor recurrence. Previous studies have shown that some inflammatory cytokines, e.g. IL-6 and VEGF, are induced in the serum and peritoneal liquids from patients during the first few days following CRC surgery, and this induction is magnified by the occurrence of a postoperative peritoneal infection [14, 20, 33]. IL-6 is tumorigenic, promotes tumor cell invasion, and serum levels of IL-6 correlate with worse prognosis in CRC [34, 35]. In turn, elevated VEGF serum levels underlie a potent angiogenic response that, as part of the inflammatory reaction to infection, may enhance the proliferation, invasion and neoangiogenesis in residual tumor cells [19, 20]. Several clinical studies point to preoperative VEGF serum levels as a putative biomarker of prognosis in, at least, subsets of patients operated of CRC [36, 37]. In this regard, we have recently found that postoperative VEGF serum concentration, along with tumor size, tumor stage and major postoperative complications, was an independent predictor of recurrence [38]. Moreover, peritoneal liquids harvested after colectomy–from cancer and non-cancer conditions–induced the in vitro migration capacity of colon cancer cell lines, and this effect correlated with increased levels of TNF-α and IL-10 in the samples [39]. Our previous study on the differential expression of cytokines in circulating leukocytes from CRC patients undergoing surgery followed by peritoneal infections also provided some potential biomarker candidates [21]. We tested some of those cytokines for which an involvement in cancer progression processes had been described and showed that some of them were also consistently induced in the THP-1 and U937 in vitro differentiated macrophages (Fig 9). Therefore, a wide range of inflammatory cytokines induced after surgery emerge as a promising source of novel biomarkers to assist in the follow up of patients operated of CRC [40].

Several protocols to differentiate monocytic THP-1 and U937 cells into macrophages have been described, including specific differentiation to M1 and M2 phenotypes based on the patterns of cytokine expression. We have chosen a differentiation protocol that was previously reported to result in the secretion of pro-inflammatory cytokines representative of a macrophage-based innate immune response–i.e., TNF-a, IL-6, IL-8, and IL-1β [22, 23], as it should more closely resemble an acute inflammatory reaction to bacterial infections. We further confirmed that our in vitro differentiation protocols also induced a M1-type cytokine expression pattern on THP-1 and U937 macrophages (Fig 4). While the β-catenin transcriptional pathway is induced on cancer cells during the course of tumor progression by protumoral, M2-type tumor-associated macrophages [41, 42], our present study is the first one, to our knowledge, to propose that an EMT-like, β-catenin/TC4-driven tumor phenotype shift is induced by macrophages on tumor cells in the context of acute inflammation. This latter notion is somewhat challenging as up to now the protumoral action of macrophages has been attributed to TAMs [30]. It will thus be one goal in future studies to validate the protumoral action of inflammatory, M1 macrophages in vivo using animal models as well as in isolated intraperitoneal macrophages from patients who undergo abdominal infections after colon cancer surgery.

We could speculate on conceivable mechanisms with which EMT may modulate the postoperative course after CRC surgery. First, EMT might confer increased drug resistance in residual tumor cells [43–47]. Moreover, the acquisition of EMT traits as a result of acute inflammation may induce tumor cell stemness thus favoring the viability of residual tumor cells, for instance in the circulating tumor cell population [48]. And finally, EMT may also facilitate the formation of distant metastasis from residual cells after surgery, which is the form of tumor recurrence most often observed after the occurrence of a postoperative intra-abdominal infection [12]. Therefore, the process of EMT induction may also constitute a source of tumor recurrence biomarkers especially relevant in the instances of acute inflammation that occur during cancer treatment.

Our present results also uncovered two aspects worth of consideration when devising in vitro assays to assess tumor recurrence biomarkers. First, several colon cancer cell lines tested by us were sensitive to the induction of invasiveness by the inflammatory medium, but another cell line, Caco2, underwent cell death as previously reported [22]. Second, different cell lines may use different signaling pathways to activate the epithelial-to-mesenchymal cell shift in response to the inflammatory medium, i.e. cell line-dependent activation of both the β-catenin/TC4 pathway (Figs 5 and 7) and EMT markers (Fig 6) and EMT transcription factors (Fig 8). Moreover, the expression patterns of cytokines expressed by the two macrophage cell lines, THP-1 and U937, were partially different but exerted a similar colon cancer cell phenotype shift (Figs 1 and 3). Therefore, our results suggest that the impact of a particular inflammatory reaction will depend on the tumor cell sensitivity to specific cytokines in each individual case. It is then plausible that combinations of several blood cytokines–elevated and/or diminished, together with the intrinsic characteristics of the tumor, will better define specific patient profiles at higher risk of recurrence. In at least the particular case of the oncological follow up after curative surgery, the THP-1/U937 model described herein together with functional assays to analyze pro-invasive and pro-angiogenic activities in postoperative blood samples [13, 20] might lead to the setting up of personalized assays to better assess the risk of tumor recurrence.

## Supporting information

S1 Fig Raw data of all the experiments mentioned in the article and their statistical analysis where applicable. (XLS)
